# Supplementary material for: Derivation of the Immortalized Cell Line UM51-PrePodo-hTERT and Its Responsiveness to Angiotensin II and Activation of the RAAS Pathway
Source: Cells. 2023 Jan 17;12(3):342. doi: 10.3390/cells12030342 (PMC9913089; doi:10.3390/cells12030342)
Supplement: Supplementary file 1 [file cells-12-00342-s001.zip › cells-2072756-supplementary - table s1, s2 and figure s1-6.pdf]

cells MDPI

Supplemental Material: Derivation of the Immortalized Cell Line-UM51-PrePodo-hTERT and Its Responsiveness to Angiotensin II and Activation of the RAAS Pathway

Lars Erichsen, Lea Doris Friedel Kloss, Chantelle Thimm, Martina Bohndorf, Kira Schichel, Wasco Wruck and James Adjaye

Table S1. antibodies.

Antigen	Company	Dilution	
		ICC	WB
α-ACTININ	Abcam (#108198)	1:200	/
ß-ACTIN	Cell Signaling Technology (3700S)	/	1:4000
HA-tag	Proteintech (51064-2-AP)	1:200	1:4000
KI67	Cell Signaling Technology (9449S)	1:200	/
NPHS1	Invitrogen (#PA5-20330)	1:200	1:1000
P53	Merck (OP43-100UG)	1:200	1:1000
pP53	Cell Signaling Technology (9283S)	1:200	/
SYNPO	Thermo Fisher Scientific (PA5-56997)	1:200	1:1000
WT1	Merck (3012219)	1:200	/
Anti-mouse HRP-labeled	Thermo Fisher Scientific #NA931	/	1:4000
Anti-rabbit HRP-labeled	Cell Signaling #7074S	/	1:1000

Table S2. RT-qPCR Primers.

Primer name	Sequence	Annealing temperature (°C)	Product length (bp)
AGTR1 s	5′- tct cag cat tga tcg ata cc -3′	60	80
AGTR1 as	5′- tga ctt tgg cta caa gca tt -3′		
AGTR2 s	5′- tat ggc ctg ttt gtc ctc at -3′	60	114
AGTR2 as	5′- cat tgg gca tat ttc tca gg -3′		
CD106 s	5′- cga acc caa aca aag gca gag ta -3′	60	84
CD106 as	5′- gag gaa ggg ctg acc aag acg -3′		
CD24 s	5′- gcg gac ttt tct ttt ggg ggg -3′	60	174
CD24 as	5′- cca gca gca gcc cca g -3′		
CD2AP s	5′- aac tca tga agc cca gga cga -3′	60	100
CD2AP as	5′- ctg atc cag atg cag ttt cac tca c -3′		
hTERT s	5′- cgg aag agt gtc tgg agc aa -3′	60	145
hTERT as	5′- gga tga agc gga gtc tgg a-3′		
KI67 s	5′- tcg tcc cag tgg aag agt tg -3′	60	144
KI67 as	5′- cag ccc cgc tcc ttt tga t -3′		
NPHS1 s	5′- gcg ggt tct gct acg atg gtg -3′	60	295
NPHS1 as	5′- caa aca cac cag cct cac ccg -3′		
P53 s	5′- cag ggc agc tac ggt ttc c-3′	60	102
P53 as	5′- cag ttg gca aaa cat ctt gtt gag-3′		
RPL s	5′- tcg aca atg gca gca tct ac -3′	60	195
RPL as	5′- atc cgt ctc cac aga caa gg -3′		
SYNPO s	5-′ ccc caa cct ctc ctc taa cc -3′	60	116
SYNPO as	5′- atg aca cag gag gca gaa gaa t -3′		
WT1 s	5′- cac agc aca ggg tac gag a -3′	60	133
WT1 as	5′- caa gag tcg ggg cta ctc c -3′		

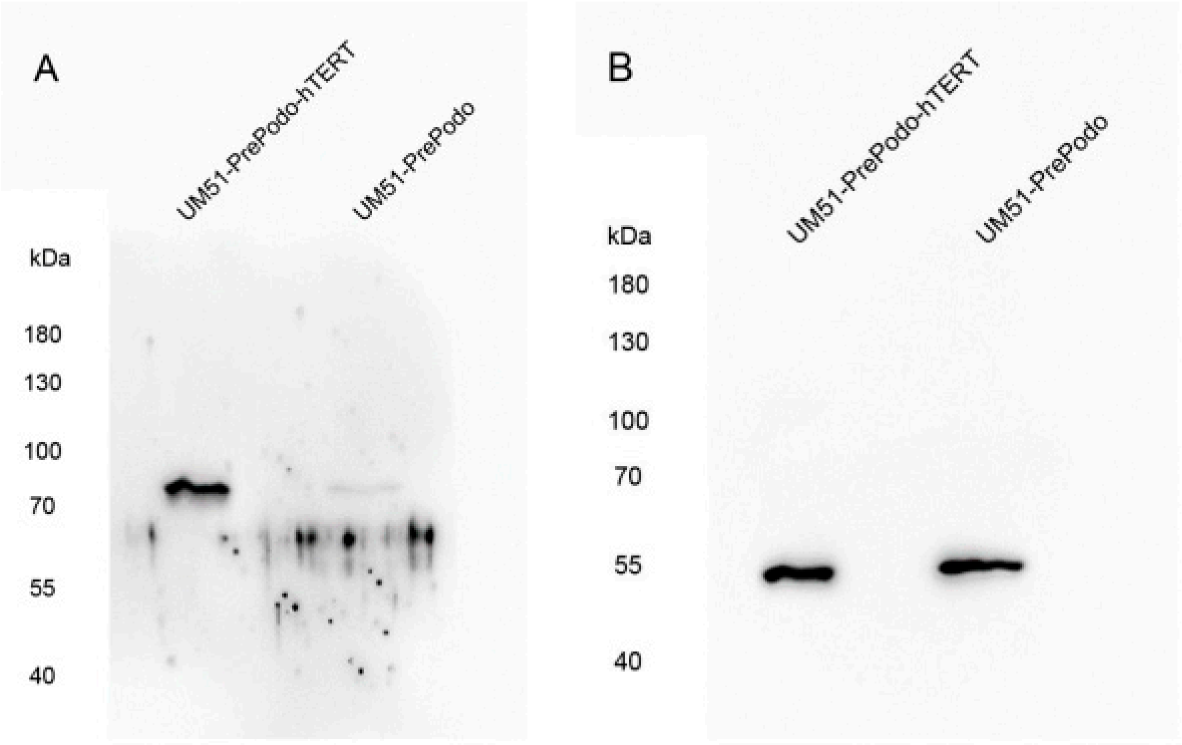

Figure S1. Complete western blot images for the detection of the HA-tag in the cell lines UM51- PrePodo-hTERT and UM51-PrePodo. (A) HA-tag, (B) β-Actin.

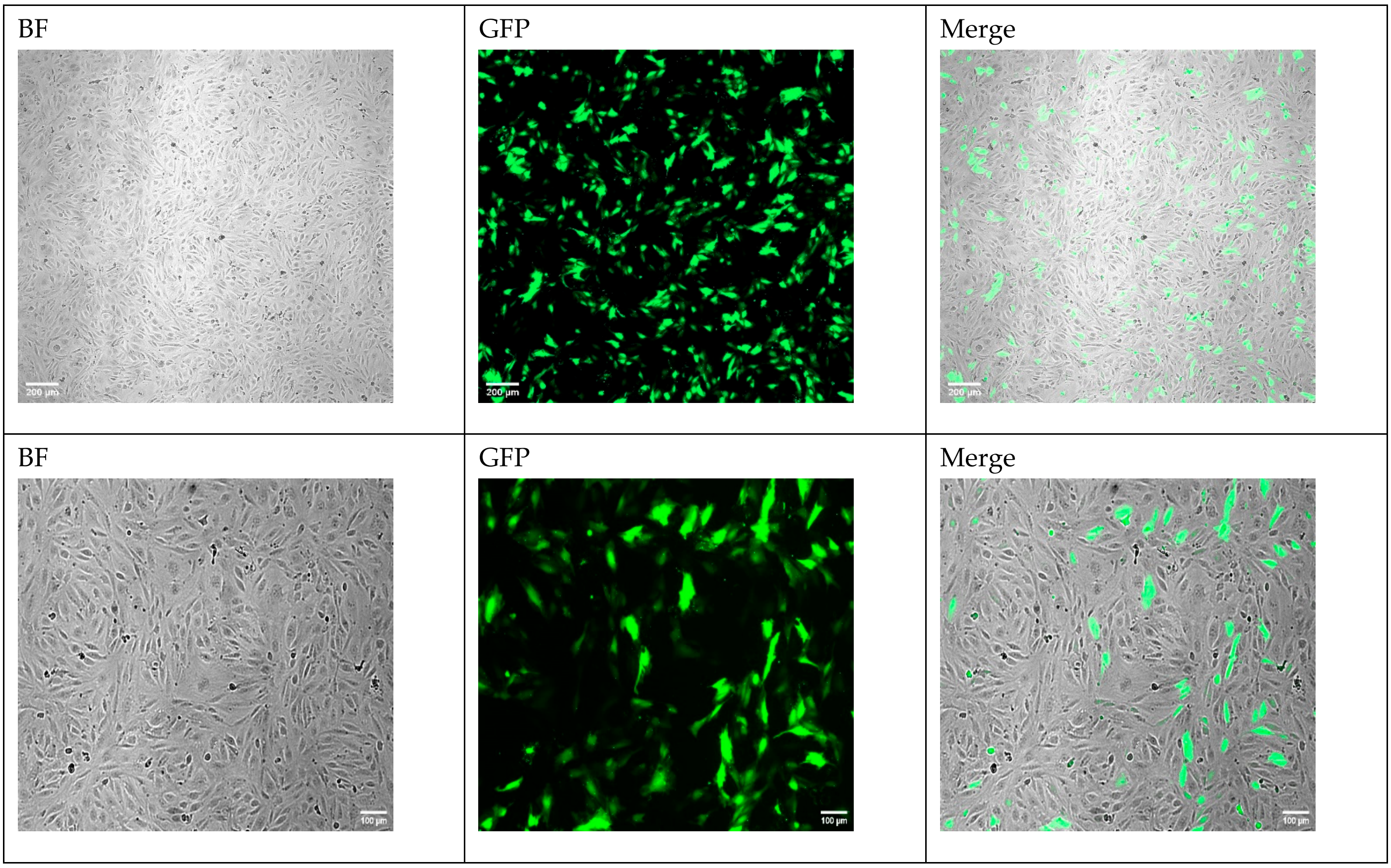

Figure S2. Transfection of UM51-PrePodo-hTERT with the TurboGFP- vector.

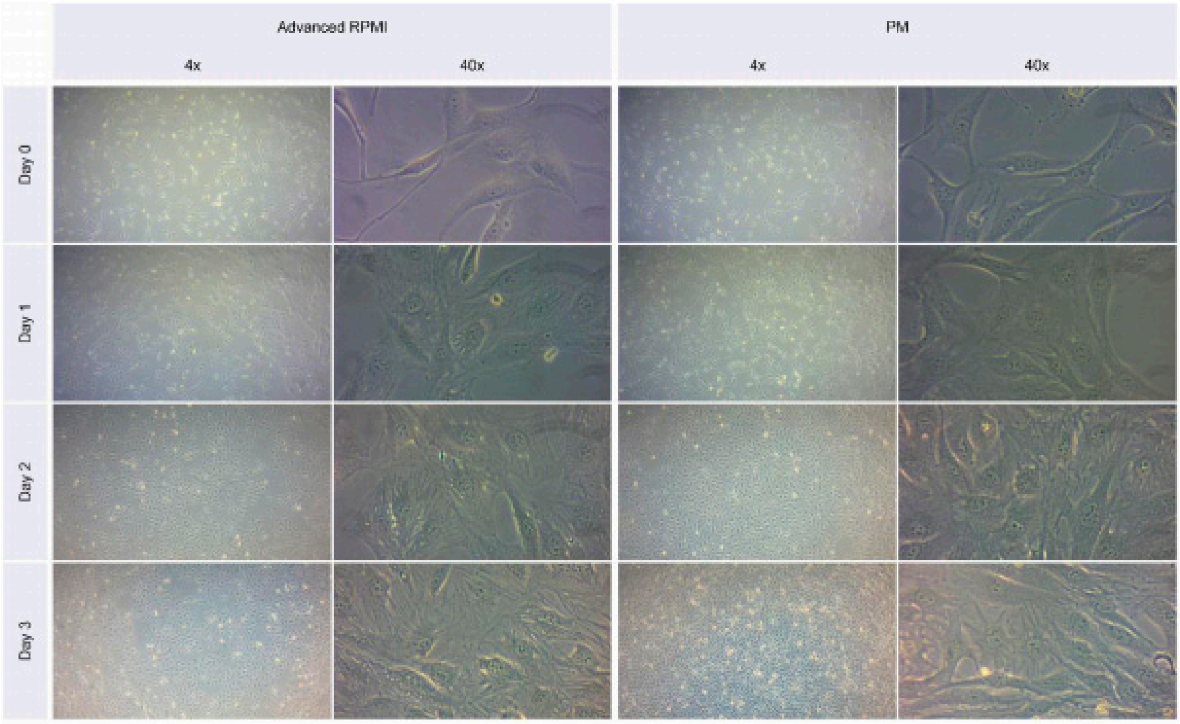

Figure S3. Morphology of UM51-PrePodo-hTERT cells cultured in Proliferation Medium (right) and Advanced RPMI (left) during the resazurin assay.

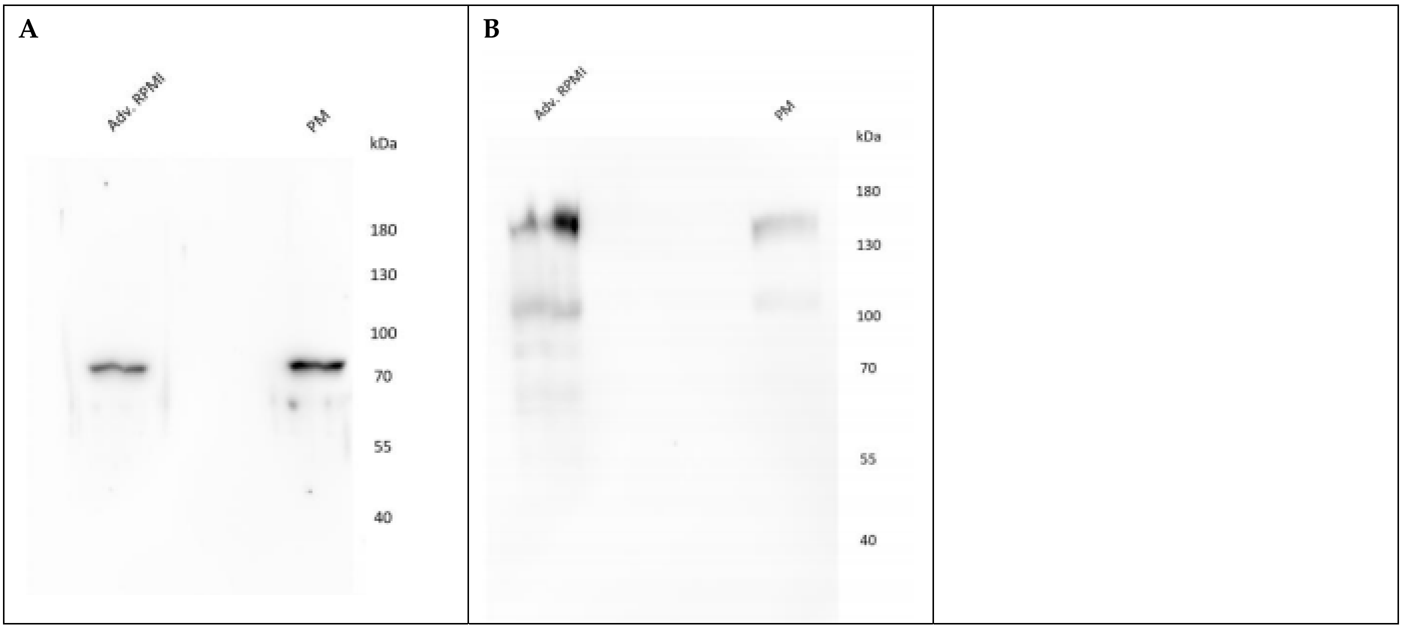

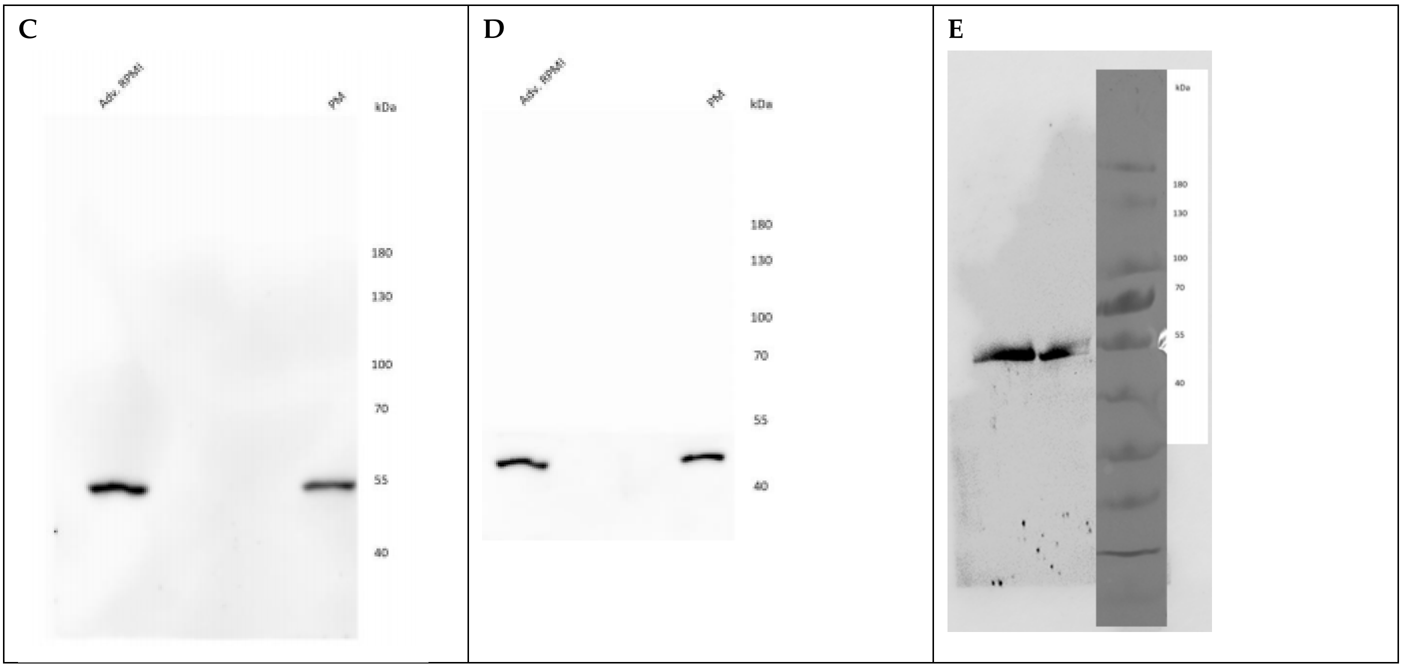

Figure S4. Complete western blot images for the comparison of UM51-PrePodo-hTERT cultured in Proliferation Medium (PM) and Advanced RPMI (Adv. RPMI). (A) HA-tag, (B) SYNPO, (C) P53, (D) β- Actin and (E) LMX1b.

A

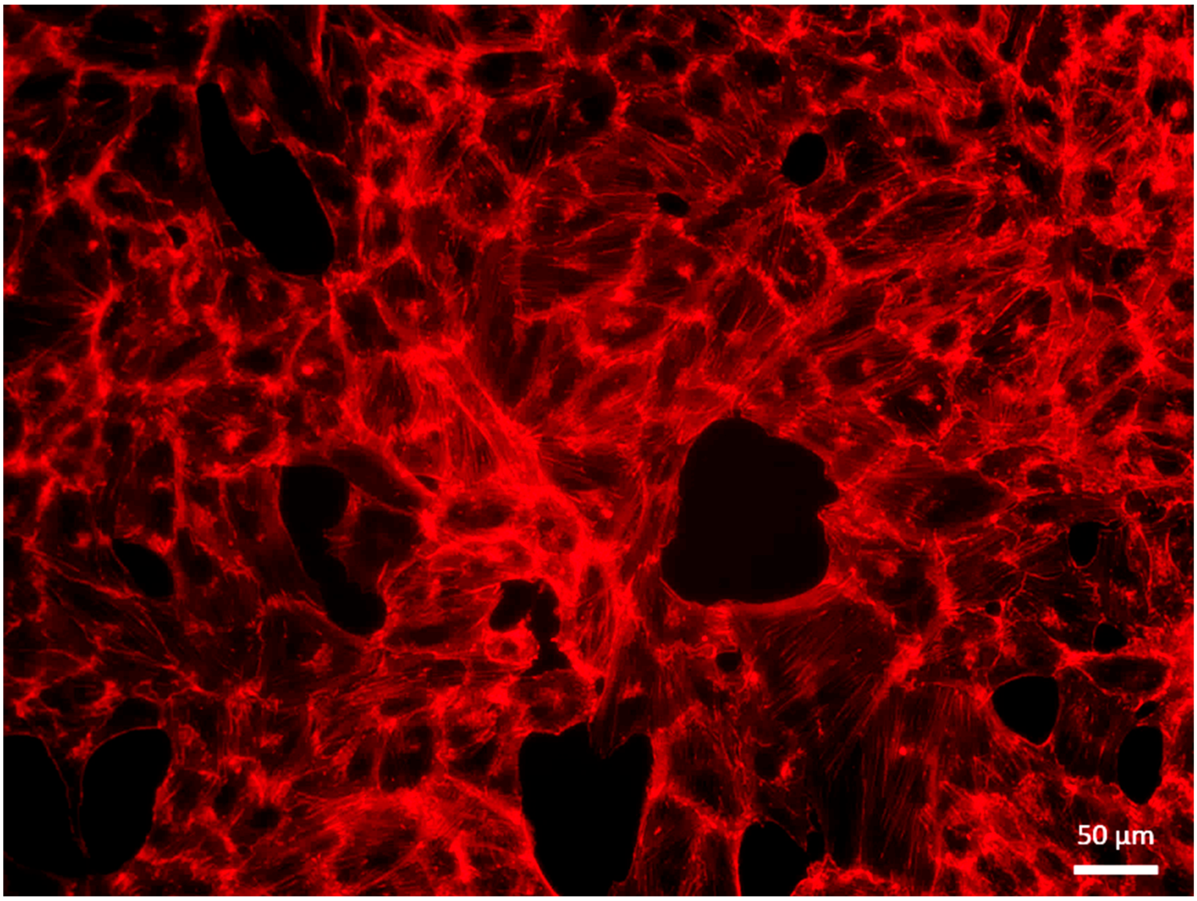

B

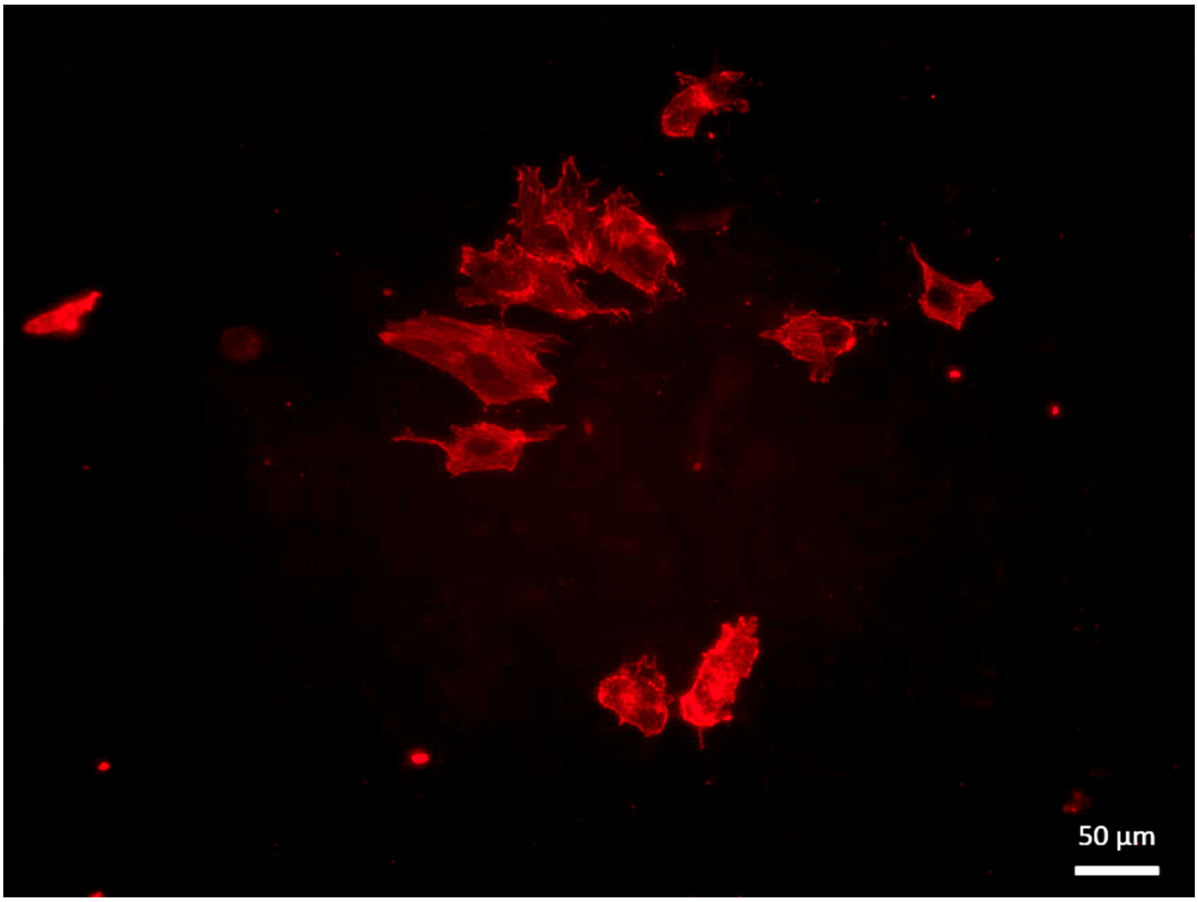

Figure S5. Higher magnification of podocyte cytoskeleton stained with α-Actinin (A). Remodeling of cytoskeleton after treatment with 100µM ANGII (B).

A

UM51 hTERT
Podocyte
15.07.2020
P3

B

UM51 hTERT
Podocyte
15.10.2021
P75

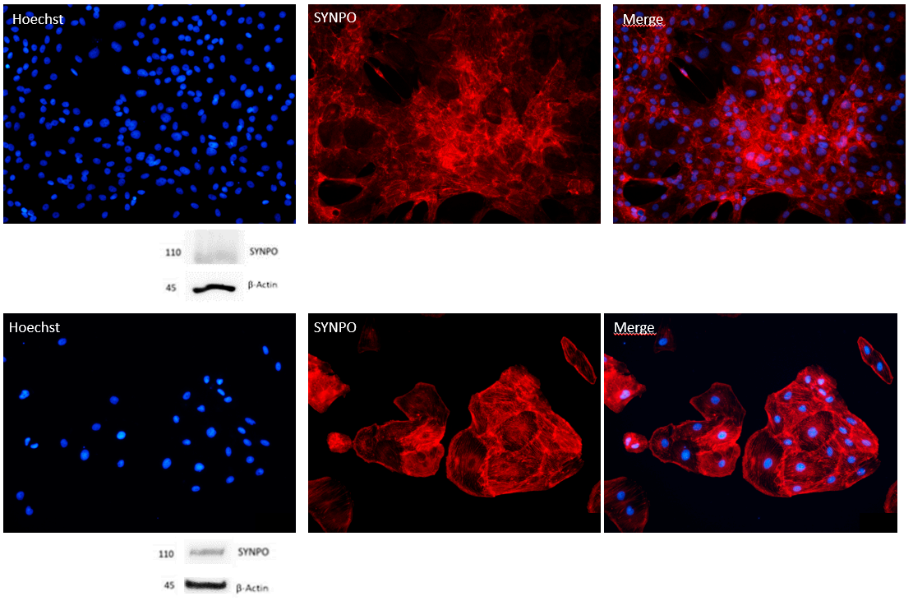

Figure S6. Comparison of UM51 hTERT passage 3 (A) and passage 75 (B). Relative protein expression of Synaptopodin was detected by Immunofluorescent based detection and Western blot analysis.
